# Integrating multiple sources of biodiversity information greatly expands the range of a rare species of Hymenoptera (Vanhorniidae)

**DOI:** 10.3897/BDJ.7.e37569

**Published:** 2019-07-16

**Authors:** Joshua Hogan, Amber I.H. Bass, Y. Miles Zhang, Barbara J Sharanowski

**Affiliations:** 1 University of Central Florida, Orlando, United States of America University of Central Florida Orlando United States of America; 2 University of Florida, Gainesville, United States of America University of Florida Gainesville United States of America

**Keywords:** Proctotrupoidea, parasitoid, Eucnemidae, *
Isorhipis
*, citizen science, museum science, databasing, distribution, *Vanhornia eucnemidarum*, digitisation

## Abstract

**Background:**

*Vanhornia eucnemidarum* Crawford is the only species of Vanhorniidae that occurs in North America. This species is rarely collected and thus the distribution is not well documented. Intending to uncover a more accurate range of this species, we assembled collection records from museums, personal collections and citizen science projects. Many of these records were non-digitised and had to be personally requested.

**New information:**

Here we expand the known distribution of *V. eucnemidarum* to include nine new provinces and states: Manitoba, Connecticut, Oregon, Mississippi, Missouri, New Hampshire, New Jersey, Texas and Wisconsin. Although Quebec has been listed as a previous locality, the recorded province was mislabelled, so Quebec is now also officially a provincial record.

## Introduction

In spite of the fact that many species on our planet remain undescribed (Scheffers et al. 2012), our in-depth knowledge of described species is often lacking in basic biology, ecology or geographic range information. With increasing efforts in the digitisation of georeferenced specimens, we expand our understanding of both described species and of overall global biodiversity (Monfils et al. 2017). There is often a wealth of information stored within smaller non-digitised collections and citizen science platforms (Ridenbaugh et al. 2018, Silvertown 2009, Skvarla et al. 2015). When collated, these sources can greatly increase knowledge on species, particularly with respect to geographic range. This highlights the importance of museums and specimen digitisation (Mehrhoff 1997), as well as the valuable part that the general public can play in expanding our knowledge of species (Ellwood et al. 2015), whether previously described or not.

**Figure 1.**
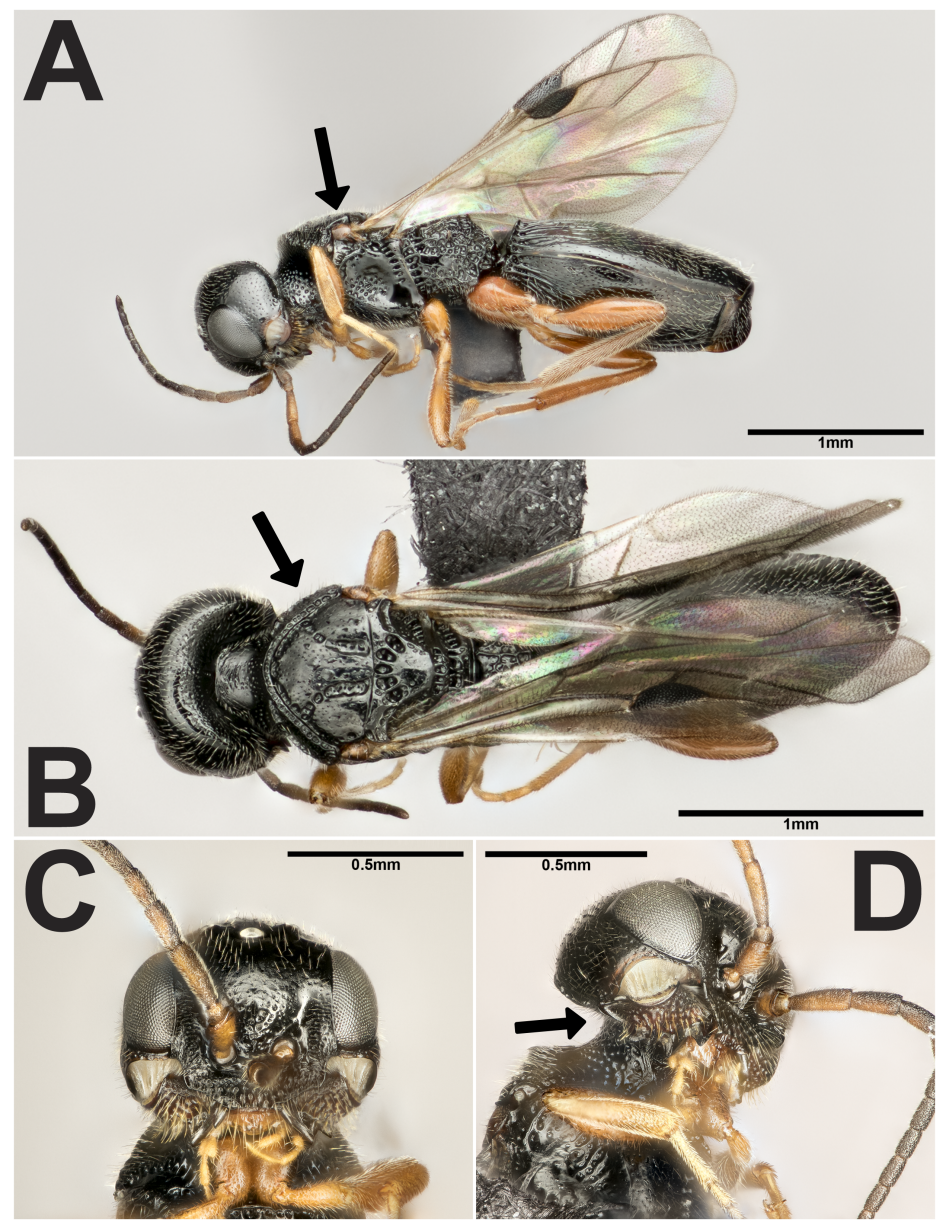
*Vanhornia eucnemidarum* female collected from Howden, Manitoba, Canada. **A.** Lateral habitus, showing brown tegula at base of wing; **B.** Dorsal habitus, showing deeply pitted mesosoma; **C.** Head anterior view; **D.** Head, antero-ventral view to show exodont mandibles.

Vanhorniidae (Hymenoptera: Proctotrupoidea, Fig. 1A) are uncommonly collected parasitoids of false click beetle larvae (Coleoptera: Eucnemidae). They can be distinguished from other proctotrupoids using a combination of characters: exodont mandibles (Fig. 1D), low attachment of the antennae (Fig. 1C), deeply pitted mesosoma (Fig. 1B) and long exserted ovipositor, projecting anteriorly from the base and housed in a ventral groove (Champlain 1922, Crawford 1909, Deyrup 1985, Haines 2008, Johnson 2017, Keim 2017, Kleiner et al. 2019, Townes and Townes 1981). Vanhorniidae contains a single genus *Vanhornia* Crawford and includes three described species, *V. eucnemidarum* Crawford, 1909, *V. quizhouensis* (He & Chu, 1990) and *V. leileri* Hedquist, 1976 (Crawford 1909, He and Chu 1990, Hedqvist 1976, Kozlov 1998). *Vanhornia eucnemidarum* is the only North American species, with a known range including the states of Florida, Georgia, Kansas, Kentucky, Maine, Maryland, Michigan, New York, North Carolina, Ohio, South Carolina, Tennessee, Virginia and West Virginia and the Canadian provinces of Ontario and Quebec (Champlain 1922, Crawford 1909, Deyrup 1985, Haines 2008, Johnson 2017, Keim 2017, Kleiner et al. 2019, Townes and Townes 1981). Along with North American records of *V. eucnemidarum*, this species has also been recorded in South Korea (Choi and Lee 2012).

Little is known about the host use and host breadth of this species. The type specimen of *V. eucnemidarum* was collected from the larval gallery of an unknown species of false click beetle (Crawford 1909). It has since been reared from the larval/pupal galleries of *Isorhipis ruficornis* (Say, 1823) found in dead maple (Brues 1927, Champlain 1922), with one record specifically from the sugar maple (Deyrup 1985). *Vanhornia eucnemidarum* may also be associated with beech (Smith 1995), another known host plant of *I. ruficornis* (Brues 1927).

Within Canada, *V. eucnemidarum* was previously recorded from Ontario and Quebec, however Townes and Townes (1981) mislabelled the Quebec specimen, which was actually found in Ontario. We collected two *V. eucnemidarum* specimens from a malaise trap in Manitoba, representing a new provincial record for Canada. This subsequently led to a search of museum records, citizen science websites and communications with professional and amateur entomologists for any unreported collections. The objective of this paper is to report all new state and provincial records of *V. eucnemidarum* to better understand its North American distribution.

## Materials and methods

Two specimens of *V. eucnemidarum* were collected by malaise trap in Howden, Manitoba (49.734996, -97.129860) between the dates of 7-14 August 2015 in a stand of trees that included maple. Additional collection records were gathered from personal communications with museums (Table 1) and collectors, online databases and the online citizen science community BugGuide (Haines 2008, Johnson 2017, Keim 2017). The majority of personal communications were facilitated through the Entomological Collections Network listserv (ECN-L). Accessed databases include iDigBio (Integrated Digitized Biocollections), DiscoverLife, SCAN (Symbiota Collections of Arthropods Network), BISON (Biodiversity Information Serving Our Nation) and GBIF (Global Biodiversity Information Facility).

**Figure 2.**
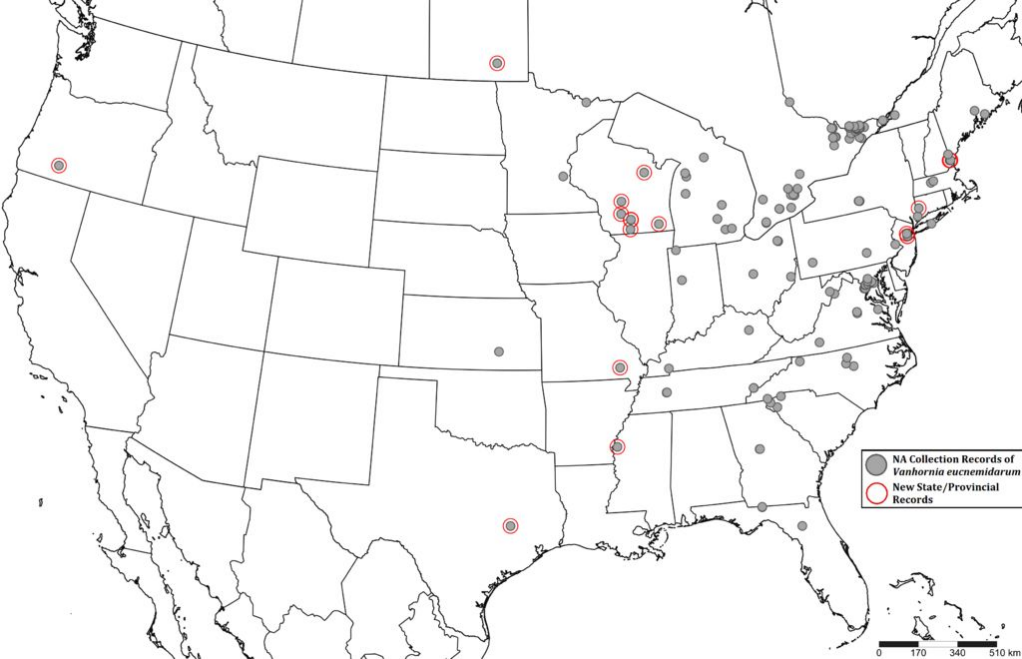
Range map of *Vanhornia eucnemidarum* in North America. Grey circles represent all North American collection records gathered during the study. Those records surrounded by a red circle represent new state/provincial records.

All locality records have been entered in Darwin Core archive format (Suppl. material 1). Any locality records that lacked geographic coordinates were input into Google Maps, generating a close approximation of the collection site (these coordinates were used for mapping, but not archived). Collection events were mapped using Simplemappr (Shorthouse 2010) with new state and provincial records indicated (Fig. 2).

All photos in this study were taken using a Canon 7D Mark II with either a Canon MP-E 65 mm F/2.8 Macro photo lens or a Mitutoyo M Plan Apo 10× objective mounted on to the Canon EF Telephoto 70-200 mm zoom lens. Multiple images were taken across numerous focal planes and combined using Zerene Stacker 1.04. Images were edited using Adobe Photoshop CC and plates were prepared using Adobe Illustrator CC.

## Taxon treatments

### Vanhornia eucnemidarum

Crawford 1909

c0ba557b-ca86-5da2-b771-3b049a0609a0

http://www.catalogueoflife.org/col/details/species/id/84c6010b1c8cef32c9a7ee8e730446b8

#### Materials

- **Type status:** Other material. **Occurrence:** catalogNumber: JBWM0378038; recordedBy: Amber Bass; sex: female; lifeStage: adult; occurrenceID: UCFC:Veuc:00000265; **Taxon:** scientificName: Vanhornia eucnemidarum Crawford 1909; kingdom: Animalia; phylum: Arthropoda; class: Insecta; order: Hymenoptera; family: Vanhorniidae; genus: Vanhornia; taxonRank: species; scientificNameAuthorship: Crawford 1909; **Location:** continent: North America; country: Canada; countryCode: CA; stateProvince: Manitoba; verbatimLocality: Howden; geodeticDatum: WGS84; **Event:** samplingProtocol: Malaise Trap; eventDate: 2015-07-14; year: 2015; month: 07; day: 14; **Record Level:** institutionCode: WRME; basisOfRecord: PreservedSpecimen
- **Type status:** Other material. **Occurrence:** catalogNumber: UCFC0528248; recordedBy: Amber Bass; sex: female; lifeStage: adult; occurrenceID: UCFC:Veuc:00000196; **Taxon:** scientificName: Vanhornia eucnemidarum Crawford 1909; kingdom: Animalia; phylum: Arthropoda; class: Insecta; order: Hymenoptera; family: Vanhorniidae; genus: Vanhornia; taxonRank: species; scientificNameAuthorship: Crawford 1909; **Location:** continent: North America; country: Canada; countryCode: CA; stateProvince: Manitoba; verbatimLocality: Howden; geodeticDatum: WGS84; **Event:** samplingProtocol: Malaise Trap; eventDate: 2015-07-14; year: 2015; month: 07; day: 14; **Record Level:** institutionCode: UCFC; basisOfRecord: PreservedSpecimen

#### Diagnosis

*Vanhornia eucnemidarum* can be distinguished from other species of *Vanhornia* by the following combination of characters: Antennal sockets inserted immediately above dorsal margin of clypeus; tegulae black to dark brown but never yellow; and rugulose metasomal striations restricted to the basal third.

## Discussion

### New records and possible host associations

Our search discovered 278 specimen records and three BugGuide photos. These data represent new records for *V. eucnemidarum* in the states of Connecticut, Mississippi, Missouri, New Hampshire, New Jersey, Oregon, Texas and Wisconsin in USA and the Canadian province of Manitoba (Fig. 2). Additionally, we present high-quality montaged photos of *V. eucnemidarum* (Fig. 1), illustrating important distinguishing features, unique to this species such as the exodont mandibles (Fig. 1D). The new records reported in this study expand the range of *V. eucnemidarum* north to Manitoba, west to Oregon and south to Texas. This vast increase in the known range of *V. eucnemidarum* may be due to rarity of collection (Deyrup 1985), lack of study or lack of recognition by non-specialists.

Though the new records of *V. eucnemidarum*, presented here, do not include host associations, they do allow for some speculation regarding the only known host, *I. ruficornis.* Several records of *V. eucnemidarum* were found to be in a state or province in which the known host *I. ruficornis* has not yet been recorded (Otto and Karns 2017). These include the states of Florida, Kentucky, Maine, Mississippi, Oregon, South Carolina, Tennessee and West Virginia, along with the Canadian province of Manitoba. This suggests that one of two things may be true: *I. ruficornis* may be present in these areas but has not yet been collected or *V. eucnemidarum* may have a wider host breadth than previously thought. If the latter is true, we suspect the host range may include other *Isorhipis* species.

The only known host plant associations of *V. eucnemidarum* are with maple and beech (Brues 1927, Champlain 1922, Smith 1995). *Isorhipis ruficornis* is a generalist on dead and rotting wood and, in addition to maple and beech, this species has been known to associate with eastern hemlock (Buck et al. 2005) and elm (Hoffmann 1942). Targeted sampling and rearing of *V. eucnemidarum* across a range of possible hosts will provide more information about the host breadth of *V. eucnemidarum* and may help predict its full range and possible biological control potential.

### Importance of museum collections and citizen science in biodiversity studies

Biodiversity studies, such as this one, assist in building more complete ranges for species, which are vital for ecological, evolutionary and applied biological research. Researchers often have limited access to these data, with an estimated 10% of specimen data stored in a digital form and even less made available online (Page et al. 2015). Only 22 (~15.4%) of the 143 novel locality records gathered during this study were previously listed in online databases. This is unsurprising as biodiversity collections and their efforts to database and digitise specimen data have historically been undervalued and underfunded, despite research showing funding for such efforts could save the government and taxpayers thousands of dollars in research costs by eliminating redundancy and allowing scientists easy access to the information they require (Lavoie 2013, Monfils et al. 2017, Suarez and Tsutsui 2004, Thiers et al. 2019).

Citizen science records provided another important source of locality data used in this study. Photos, uploaded through citizen science projects and social media websites such as BugGuide, Flickr and iNaturalist, are becoming frequent sources of legitimate taxonomic records (Ridenbaugh et al. 2018, Dickinson et al. 2012, Skvarla et al. 2015, Silvertown 2009, Thiers et al. 2019). This further demonstrates the importance of the unique natural history observations citizens provide researchers.

### Conclusions

This study has updated the distribution records of *V. eucnemidarum*, using a combination of museum, citizen science and digitised records. Given the large increase in range discovered through this study, it is possible that this species is present throughout North America. We hope this paper will aid in recognition of *V. eucnemidarum* by curators and naturalists, which will further our understanding of this enigmatic family of parasitoid wasps.

## Supplementary Material

681c9921-66bc-5f3f-9b21-a6f5ab2021b1 10.3897/BDJ.7.e37569.suppl1 Supplementary material 1 *Vanhornia eucnemidarum* NA Records Data type: Occurrences
Brief description: This datasheet provides an accessible way to search collection records gathered during this study.
File: oo_312013.xlsx https://binary.pensoft.net/file/312013 Joshua Hogan

XML Treatment for Vanhornia eucnemidarum

## Figures and Tables

**Table 1.** Listed are all collections referenced in this manuscript and supplemental along with their associated acronyms

**Acronym**	**Name of Collection**
AMNH	American Museum of Natural History
CAS	California Academy of Sciences
CNCI	Canadian National Collection of Insects
DEBU	University of Guelph
LEMQ	Lyman Entomological Museum
FSCA	Florida State Collection of Arthropods
MCZC	Museum of Comparative Zoology
MEM	Mississippi State University
MSUC	Michigan State University
NCSU	North Carolina State University
PMAE	Royal Alberta Museum
PMNH	Peabody Museum of Natural History
INHS	Illinois Natural History Survey
OSUC	C.A. Triplehorn Insect Collection
QMOR	Collection Entomologique Ouellet-Robert
RMNH	Naturalis Biodiversity Centre
ROME	Royal Ontario Museum
SEMC	Snow Entomological Museum
TAMU	Texas A & M University
UCFC	University of Central Florida
UMMZ	University of Michigan
UMSP	University of Minnesota
UNHC	University of New Hampshire
WIRC	University of Wisconsin
WRME	Wallis Roughley Museum of Entomology
